# Sunflower Cake from the Biodiesel Industry in the Diet Improves the Performance and Carcass Traits of Nellore Young Bulls

**DOI:** 10.3390/ani12233243

**Published:** 2022-11-23

**Authors:** Vinicius da Silva Oliveira, Analivia M. Barbosa, Ederson A. de Andrade, Gercino F. Virginio Júnior, Thiago V. C. Nascimento, Anny Graycy Vasconcelos de Oliveira Lima, Ricardo W. D. Portela, Jarbas M. da Silva Júnior, Elzânia S. Pereira, Leilson R. Bezerra, Ronaldo L. Oliveira

**Affiliations:** 1 Department of Animal Science, Federal University of Bahia, Salvador 40170-110, BA, Brazil; 2 Campus Sertão, Federal University of Sergipe, Nossa Senhora da Glória 49680-000, SE, Brazil; 3 Animal Science Department, Federal University of Ceará, Fortaleza 60020-181, CE, Brazil; 4 Department of Animal Science, Federal University of Campina Grande, Patos 58700-000, PB, Brazil

**Keywords:** beef cattle, by-product, *Helianthus annuus*, microbial proteins, nitrogen

## Abstract

**Simple Summary:**

Sunflower cake is a by-product of biodiesel production, and its nutritional characteristics, such as crude protein content (249 g/kg DM), contribute to its potential for use in ruminant feed. This study evaluated the potential of partially replacing traditional sources (soybean meal and ground corn) with sunflower cake by examining performance, nitrogen excretion, ingestive behavior, and carcass quality. Our results demonstrate the potential benefit of using sunflower cake up to 90 g/kg DM in the diet of young bulls, especially in countries where this by-product is available.

**Abstract:**

We aimed to determine the optimal inclusion level of sunflower cake (0, 90, 180, and 270 g/kg total DM) as a partial replacement of soybean meal and corn ground in young bulls’ diets by examining nutrient intake and digestibility, ingestive behavior, nitrogen balance, metabolic serum profile, growth performance, and carcass traits. Thirty-two intact Nellore bulls (BW 374 ± 42.5) were distributed in a completely randomized design. The experiment lasted 90 days. The final BW of the animals was 515.25 ± 24.7. There was a linear decrease effect in the intake of DM, crude protein and nonfibrous carbohydrates, eating and rumination efficiency, N-urinary, N-total excretion, and blood urea nitrogen. Sunflower cake did not affect the NDF digestibility, nitrogen (N)-fecal excretion, blood metabolites, *Longissimus lumborum* muscle area, or subcutaneous fat deposition. There were linear and quadratic effects on the eating and rumination time, microbial protein production and efficiency, gamma-glutamyl transferase and cholesterol serum concentrations, and muscle carcass tissue. There was a quadratic effect on ether extract intake, final BW, and total gain with the inclusion of sunflower cake in the young bull’s diet. The replacement of soybean meal and corn ground with sunflower cake at the level of 90 g/kg of DM in the diet of young bulls is recommended because it reduces the DM intake and digestibility, increases microbial protein synthesis and muscle tissue deposition, and consequently improves the performance, feed efficiency, and carcass traits.

## 1. Introduction

One of the many ways to increase a productive systems’ economic viability is to change the diet–ingredient composition by using feed alternatives, such as the residues from biodiesel extraction and agro-industrial oil production [1,2,3]. The use of these residues, especially in replacement of ground corn and soybean meal, are used as alternative energy and protein sources, especially in ruminant feed, allowing not only a reduction in production costs but also the ecological use of a residue, that until then had no purpose [4,5,6]. Moreover, it is necessary to remember that corn and soy and their by-products are common ingredients in the human diet, which generates feed–food competition [7,8].

Biodiesel production generates many by-products (cakes and refusals) available for feeding ruminants [8,9]. One example, sunflower cake, is obtained after the mechanical extraction of sunflower (*Helianthus annuus*) oil [10], has high concentrations of lipids (160 g/kg on a DM basis) and proteins (249 g/kg on a DM basis) [11], and has been recommended for total or partial replacement of the soybean meal and corn components [11,12] in feed for goats [13], lambs [14] and steers [3,15].

The protein present in sunflower cake is highly degradable in the rumen, and its incorporation improves the use of N and the efficiency of microbial metabolism [7,16]. However, when supplied in high quantities, sunflower cake can increase the diet ether extract (EE) content, which is associated with the decrease in the total neutral detergent fiber (NDF) digestibility due to high-fat content that affects microorganism’s metabolism [17,18,19]. Thus, affecting animal performance by decreasing it [11,12,20]. Therefore, it is necessary to precisely evaluate intake, digestibility, and animal performance to characterize the alternative feed.

Thus, we hypothesized that partial replacement of corn ground and soybean meal by sunflower cake could improve animal performance without affecting the nutrient intake. Therefore, we aimed in this study to determine the optimal inclusion level of sunflower cake in the diet of young bulls, considering its effect on nutrient intake, ingestive behavior, digestibility, nitrogen balance, microbial production efficiency, the serum metabolic profile, animal performance, and carcass characteristics.

## 2. Materials and Methods

### 2.1. Location and Ethical Approval

The experiment was conducted at the Federal University of Bahia (UFBA) following Institutional Animal Use Ethics Committee (protocol number 02/2014).

### 2.2. Animals, Experimental Design, and Experiment Duration

Thirty-two young male Nellore bulls (age = 24 months) with initial body weights (BWs) of 374 ± 42.5 kg were blocked by initial BW and randomly assigned to one of four levels of sunflower cake inclusion (0, 90, 180, and 270 g/kg of dry matter (DM)) in partial replacement of soybean meal and corn ground (*n* = 8 per treatment). The trial lasted 105 days, including 15 days for adaptation to the individual pens and experimental diets.

During the adaptation period, the animals were identified, weighed, treated with oral ivermectin (Ranger LA^®^, Vallée, Uberlândia, Brazil), and distributed into individual covered pens (2 × 4 m) with free access to feeders and drinking water.

### 2.3. Diets and Chemical Composition

Diets were composed of 400 g/kg Tifton-85 bermudagrass hay (*Cynodon* spp.) as roughage and 600 g/kg concentrate (corn ground, soybean meal, urea, ammonium sulfate, a mineral mixture, and sunflower cake). The diets were formulated according to National Research Council (NRC) [21] and contained 150 g/kg crude protein (CP), allowing for an estimated average daily gain (ADG) of 1.5 kg/d. Animals were fed a total mixed ratio (TMR) twice daily at 9:00 and 16:00 h. The offer was estimated to allow 10% of refusals, which were removed and weighed against allowing the adjustments on the amount offered daily. The samples of the diet ingredients, TMR, and refusals were collected weekly (during the entire trial, 13 weeks) and frozen (–20 °C) for chemical analysis (Table 1 and Table 2).

The samples collected were dried at 55 °C for 72 h, ground in a Wiley mill (Tecnal, Piracicaba, São Paulo, Brazil), in a 1 mm sieve and stored sealed in airtight plastic bowls (ASS, Ribeirão Preto, São Paulo, Brazil) for further analysis to determine the contents of DM (method 930.15), crude protein (CP; method 968.06), EE (method 954.05), and ashes (method 942.05) following the AOAC [22].

The NDF and acid detergent fiber (ADF) contents were determined, followed by Van Soest et al. [23]. The acid detergent lignin (ADL) contents were determined using ADF residue treated with 72% sulfuric acid [22]. The residue was incinerated in an oven at 600 °C for 4 h and corrected for the protein content (_ap_NDF). The neutral detergent insoluble nitrogen (NDIN) and acid detergent insoluble nitrogen (ADIN) values were obtained following the Licitra et al. [24]. The hemicellulose and cellulose contents were calculated by subtracting the NDF content from the ADF content and the ADF content from the lignin content, respectively.

Nitrogen fractions were divided into A + B1 + B2, B3, and C, following the methodology by Licitra et al. [24]. Carbohydrates fractions were divided into A + B1, B2, and C, according to Sniffen et al. [25]. Nonfibrous carbohydrates (NFC) were determined following Hall [26].

### 2.4. Intake and Ingestive Behavior

Nutrient intake was determined by subtracting each nutrient content measured by the refusals from the content of each nutrient in the feed. The chemical composition of the effectively ingested diet was estimated by dividing the intake of each nutrient by DM intake, and the quotient was multiplied by 100.

To evaluate ingestive behavior, individual animal observations were performed on days 30 and 60, at 5 min intervals over 24 h, according to Bateson and Martin [27]. The eating rate (ER) and rumination rate (RR) were calculated, and these variables were further calculated from the ratio of DM or NDF (kg) to time spent eating or ruminating (h).

### 2.5. Nutrients Digestibility and Nitrogen (N) Balance

Fecal and urine samples were collected daily between days 36 and 42 of the experimental period. To collect feces, appropriate canvas bags were attached to the animals using nylon strips to reduce the inconvenience for the young bulls. The feces were accumulated in the bags, and collections occurred at two times: between 9 and 11 h, and later between 16 and 18 h. Fecal samples were collected after homogenization of the feces, and about 50 g of fresh material was collected [28]. After the collections, the bags were emptied. Then, the fecal samples were mixed into a pooled sample, weighed, identified, and stored at −15 °C for further analyses. Subsequently, the fecal samples were immediately oven-dried at 55 °C for 72 h, ground in a Wiley mill (model 3, Arthur H. Thomas, Philadelphia, PA, USA) in a 2 mm sieve. The contents of DM, CP, EE, NFC, NDF, and TDN were analyzed as previously described. Indigestible neutral detergent fiber (_i_NDF) was used as an internal marker to estimate fecal excretion [23]. Thus, diet, feces, and refusal samples (20 mg DM/cm^2^) were placed in polypropylene bags (nonwoven) [29] and incubated for 288 h in the rumen of two young fistulated bulls (372 ± 35.1 kg and 23 months of age). The residue after the incubation was washed until the water became clear and dried under forced ventilation at 55 °C for 72 h. The _i_NDF content was determined following the Van Soest et al. [23]. To estimate fecal production (kg DM/d), the total indicator ingested was divided by the indicator concentration in the feces.

The digestibility (the amount of the diet which was digested, and was not lost through the feces) coefficients (DC) of DM, CP, EE, NDF, and NFC were calculated by using the equation, with the values in DM content:

DC = (kg of the portion ingested − kg of the portion excreted)/(kg of the portion ingested) × 100. (1)

The total digestible nutrient (TDN) intake was calculated according to Sniffen et al. [25] using the equation

TDN_I_ = (CP_I_ − CP_F_) + 2.25 (EE_I_ − EE_F_) + TC_I_ − TC_F_), (2)

where _I_ represents the nutrient intake, and _F_ refers to nutrients excreted in the feces, respectively. The concentrations of TDN were obtained using the equation

TDN (g/kg) = (intake of TDN/intake of DM).(3)

During the digestibility trial, about 2–4 samples/d were collected from each animal in vessels containing a 20 mL solution of 2% H_2_SO_4_. Samples were collected for 4 days (at any time of day), and subsequently, these samples were pooled per animal at the end of the experimental trial. Thus, each animal was a replicate [30]. Urine was obtained using disposable cups to minimize microbial contamination and was filtered to remove solid particles. The samples were stored frozen at −10 °C before analysis.

The urea concentration in blood serum and the creatinine concentration in urine were obtained by a colorimetric enzymatic assay (Labtest^®^ Diagnóstico SA, Minas Gerais, Brazil) in an AutoAnalyzer II (In Vitro Diagnostica, Itabira, MG, Brazil). Allantoin and uric acid contents in urine were estimated using colorimetric methods [31], and the total N content was also estimated [22].

The total urine volume was estimated from the creatinine concentration in the urine upon excretion per unit of BW:

CE = 32.27 − 0.01093 × BW, (4)

where CE is the concentration of creatinine excreted daily (mg/kg of BW) [32]. Purine derivative excretion was calculated as the sum of allantoin and uric acid excreted in the urine. The purine absorption was calculated using the following equation [33]:

AP = (PD − 0.385 × BW^0.75^)/0.85 (5)

where AP is the rate of purine absorption (mmol/d), PD is the rate of purine derivative excretion (mmol/d), 0.85 is the proportion of absorbed purines recovered as purine derivatives in the urine (mmol/mmol), and 0.385 is the endogenous purine derivative excretion in the urine per unit of metabolic BW (mmol) [31].

The microbial synthesis of nitrogenous compounds was estimated as a function of the absorbed purines and the N_RNA_:N_TOTAL_ ratio in the microorganisms [31]:

Nmicr = (70 × AP)/(0.83 × R × 1000),(6)

where Nmicr is the microbial nitrogen flow in the small intestine (g/day), R is the N_RNA_:N_TOTAL_ ratio in the microorganisms (mg/mg), 70 is the N content in purines (mg/mol), and 0.83 is the intestinal digestibility of the microbial purines (mg/mg). The microbial synthesis efficiency (g Nmicr/100 g TDN) was determined by dividing the microbial protein production by the TDN intake. The N retained was determined using the following equation:

N retained (g/d) = N-intake − N-total excretion.(7)

Total N excretion was calculated from the fecal N excretion and N-urinary excretion sum. The urinary N, N-fecal, and N-total excretion were expressed as g/100 g N-intake.

### 2.6. Blood Metabolites

The blood samples (e.g., 10 mL) were collected in tubes without anticoagulants before morning feeding on experimental day 30 by jugular venipuncture. After clotting, the blood serum was centrifugated at 2000× *g* for 10 min (Centrilab^®^ model CE3001, São Paulo, Brazil). The serum was stored in a freezer at −20 °C for further analysis.

The blood parameters were analyzed in a semiautomatic biochemical analyzer (BioPlus 2000^®^, São Paulo, Brazil), following specific methods (Labtest^®^ Diagnostic SA, Minas Gerais, Brazil) to determine albumin (Ref. 19), the total protein concentration (Ref. 92–250), glucose (Ref. 85), triglycerides (Ref. 87), total cholesterol (Ref. 76), alanine aminotransferase (ALT; Ref. 1008), aspartate aminotransferase (AST; Ref. 109), and gamma-glutamyltransferase (GGT; Ref. 105). The dosed total bilirubin was determined by the Sims–Horn method through diazotization and the formation of red azobilirubin. The globulin concentration was calculated by the difference between the total protein and serum albumin concentrations. The albumin: globulin ratio was also calculated.

### 2.7. Performance and Carcass Traits

The young bulls were individually always weighed before morning feed delivery, at the beginning of the experiment (initial BW), every 30 days to determine the ADG and feed efficiency (kg ADG:kg DMI ratio), and before slaughter to obtain the final BW and total weight gain (TWG). The ADG was calculated as the difference between the initial and final BWs of the animals divided by the number of days in the trial period.

The bulls fasted for 16 h before the slaughter. Slaughter was carried out in a commercial slaughterhouse. All animals were stunned (Dal Pino, Santo André, SP, Brazil), bled, skinned, and eviscerated.

The head and feet were removed, and the carcasses were weighed to determine the hot carcass weight (HCW) and hot carcass yield (HCY) with the equation

HCY = [HCW/slaughter BW (SBW)] × 100. (8)

The carcasses were placed in a cold chamber (4 °C) for 24 h, and subsequently weighed for determination of the cold carcass weight (CCW) and cold carcass yield (CCY) following the equation:

CCY = [CCW/SBW] × 100. (9)

The carcass yield was evaluated in all the carcass. The carcass length was measured on the inside of the left half of the carcass with a measuring tape graduated in centimeters as the distance between the base of the neck and the tail.

The carcass compactness index was obtained by dividing the CCW by the internal length. After cooling for 24 h, each carcass was measured in the *longissimus lumborum* muscle area (LMA, cm^2^). On the left side of each carcass, a cross-section was made between the 12th and 13th ribs, exposing the *longissimus lumborum* muscle, which was traced with an A4 75-micron transparency sheet (P/ink-jet, Kalunga, São Paulo, Brazil); this area was subsequently measured using a digital planimeter (DIGIPLAN 300/301, Herbert Kreite, Bonn, Germany). The subcutaneous fat thickness (SFT) was measured in mm at the cutting area between the 12th and 13th ribs on the *longissimus lumborum* muscle. The reference for the SFT measurement was a point corresponding to three-quarters of the section width from the transverse process of the thoracic vertebrae.

Samples of the section between the 9th and 11th ribs (section HH) were collected and dissected, and the proportions of muscle (meat), adipose tissue, and bones contained therein were estimated based on the proportions of these components in section HH [34].

### 2.8. Statistical Analysis

Data were analyzed using mixed models (PROC MIXED) in Statistical Analysis Systems (SAS) for a completely random design with four treatments (sunflower cake inclusion levels of 0, 90, 180, and 270 g/kg DM), each with eight replicates (eight animals per treatment, totaling 32 animals used in the trial). The animals were used as the experimental unit and were used as a random effect and the treatments as the fixed effect. When analyzing the ADG and ADG:DMI data, the initial BW was used as a covariate for statistical analysis using the following model:

Y*ij* = *µ* + T*i* + *β*(W*ij* − W) + e*ij*, (10)

where Y*ij* = the observed value of the dependent variable (ADG: DMI) in animal j receiving treatment *i*; *μ* = the general mean; T*i* = the fixed treatment effect *i* (*i* = the effect of the sunflower cake level: 0, 90, 180 and 270 g/kg DM); *β* = the regression coefficient relative to covariate W*ij*; W*ij* = the covariate effect (initial BW of animal j receiving treatment *i*); and e*ij* = the effect of the experimental error. For the other data (from 32 animals), the following model was used:

Y*ij* = *μ* + s*i* + e*ij,* (11)

where Y*ij* = observed value of the dependent variable in animal *j* receiving treatment *i*; *μ* = the overall mean; s*i* = the effect of the sunflower cake level; and e*ij* = the effect of the experimental error. The means were calculated using the PROC MIX of SAS, and the linear, quadratic, and cubic effects were evaluated. As the cubic effect was not significant for any studied variable, we chose not to include it in the tables. Significance was considered when *p* ≤ 0.05.

## 3. Results

### 3.1. Intake, Ingestive Behavior, Apparent Total Tract Digestibility of DM, CP, EE, NFC, NDF, N Balance, and Serum Metabolites

The inclusion of sunflower cake in the diet of young bulls did not linearly (*p* > 0.05) or quadratically (*p* > 0.05) affect the intake of apNDF (kg/d and g/kg BW) and TDN in young bulls (Table 3). However, the EE intake (kg/d) linearly increased (*p* < 0.001), while the CP (*p* = 0.004) and DM in kg/d or g/kg BW, NFC (*p* < 0.001) intake (kg/d) linearly decreased with the increase in the level of sunflower cake replacement of soybean mean and corn ground in the diet. The amount of EE and apNDF F effectively ingested increased linearly (*p* < 0.001), and the CP (*p* = 0.002) and NFC effectively ingested decreased linearly (*p* < 0.001) due to sunflower cake inclusion.

There were both linear (*p* < 0.001) and quadratic (*p* = 0.033) increases in the rumination time and a linear increase (*p* = 0.014) in eating time as the amount of sunflower cake replacing soybean meal and corn ground in the diet of young bulls increased. In contrast, the time spent idling and eating efficiency rate (kg DM/h; kg apNDF/h) linearly (*p* < 0.05) and quadratically (*p* < 0.05) decreased with the increasing inclusion of sunflower cake in the diet of young bulls. The eating time was not affected linearly (*p* = 0.142) or quadratically (*p* = 0.553).

The inclusion of sunflower cake promoted only a linear decrease in the apparent total tract digestibility of the DM (*p* < 0.001) and apNDF (*p* < 0.001) in young bulls (Table 4). The CP, EE, NFC digestibility, and TDN were affected neither linearly (*p* > 0.05) nor quadratically. There was a linear decrease (*p* < 0.001) in the N-intake, N-urinary (*p* < 0.001) and total N excretion (*p* = 0.002) as g/d and in the N-urinary (*p* < 0.001) and N-total excretion (*p* = 0.003) as g/100 g N-intake with the increasing inclusion of sunflower cake in the diet of young bulls. However, neither the N-fecal excretion in g/d (*p* = 0.498) and as g/100 g N-intake (*p* = 0.566) nor the N-retained (*p* = 0.92) in young bulls were affected linearly or quadratically by the sunflower cake inclusion. The microbial protein production and the efficiency of this production linearly and quadratically increased, respectively, with increasing sunflower cake inclusion.

There was neither linear (*p* > 0.05) nor quadratic effects of sunflower cake inclusion in the diet of young bulls on the serum concentrations of total protein, albumin, globulin, triglycerides, AST, ALT, and the albumin:globulin ratio. The blood urea nitrogen (BUN) concentration linearly decreased (*p* = 0.051), the GGT concentration both linearly (*p* = 0.034) and quadratically (*p* = 0.023) increased, and the total cholesterol (*p* = 0.042) and total bilirubin (*p* = 0.013) linearly increased with increasing sunflower cake inclusion.

### 3.2. Performance and Carcass Traits

The final BW, TWG, ADG, feed efficiency (ADG:DMI ratio), HCW, and CCW quadratically increased (*p* < 0.05), with the highest values observed for the inclusion of 90 g/kg DM sunflower cake in place of soybean meal and corn ground in the young bull diet. However, the HCY, CCY, cooling loss, carcass length, carcass compaction index, LMA, and SFT of young bulls were affected neither linearly (*p* > 0.05) nor quadratically by sunflower cake inclusion (Table 5).

There was a linear increase in the carcass length (*p* = 0.093) of young bulls with increasing sunflower cake inclusion in the diet. The bone tissue of carcasses linearly (*p* = 0.084) and quadratically (*p* = 0.083) tended to decrease. In contrast, the muscle tissue linearly (*p* = 0.072) and quadratically (*p* = 0.053) increased, and the muscle/bone ratio increased linearly (*p* < 0.001) and quadratically (*p* = 0.012). The fat content (*p* = 0.402) and muscle:fat ratio (*p* = 0.192) of the carcass tissue of young bulls were not affected linearly (*p* > 0.05) nor quadratically by sunflower cake inclusion in the diet.

## 4. Discussion

The inclusion of sunflower cake in the young bulls’ diets reduced DMI linearly due to increased lipid content in the diet and, consequently, reduced the CP intake [15,19,21] linearly. However, despite this effect, the performance variables showed a quadratic effect. It was negatively affecting the DM and _ap_NDF digestibility and may have occurred due to the lipids’ capacity to inhibit the microorganism fixation and degradability of the feed [21]. The lipids can affect the microorganism’s fixation to the fiber in a variety of ways, such as by physically covering dietary fiber or by preventing microorganism attack from the modification of the microbial population in the rumen, through toxic effects caused by the active effects of dietary fat on the surface of microorganism membranes [11,35]. However, we did not evaluate the ruminal bacterial community to understand the possible effects of sunflower cake.

Young bulls receiving more sunflower cake spent more time eating and ruminating, probably because of the B2 fraction (slower degradation rate) of carbohydrates that increased (327 to 366 g/kg DM) [36,37]. Consequently, the time spent idling, eating, and ruminating efficiency rate (kg DM/h) were reduced, which corroborate with the data found by other researchers, which found that the increase in NDF content led to an increase in the feed efficiency [11,36,37].

The decrease in N intake explains the reduced excretion of N-urinary, N-total excretion, and BUN, which improves the Nmicr production by microorganisms and, consequently, microbial production efficiency [16,38]. Agy et al. [7] and Gonzaga et al. [11] observed that sunflower cake has CP with high ruminal degradability and rumen-undegradable protein with high digestibility. In addition, these components promote a reduction in BUN and urinary N excretion, which demonstrates a more efficient use of N compounds from the diet, allowing to state that there was a better balance between N and energy [12,15,18,36].

We analyzed blood enzymes related to liver function to see if there would be an overload of fat metabolism due to the inclusion of sunflower cake in the animals’ diets. The use of sunflower cake reduced the blood concentration of GGT enzymes; however, we do not believe that GGT is related to the addition of sunflower cake because the AST and ALT enzymes did not change. Antunović et al. [39] also observed GGT reduction in lactating cows. The inclusion of sunflower cake in replacement of soybean meal and corn ground increased the serum cholesterol by 78 mg/dL but did not influence the serum triglyceride concentrations, with a mean value of 101 mg/dL.

The inclusion of sunflower cake at a 90 g/kg DM level promoted higher muscle deposition and improved performance, feeding efficiency (ADG:DMI ratio), and carcass weight. The maximum muscle:bone ratio was observed at the level of 90 g/kg sunflower cake inclusion because the muscle carcass tissue increased, and the bone carcass tissue decreased. These results are explained by the increase in the lipids intake with the increasing inclusion of sunflower cake that influenced the dietary protein use efficiency, leading to greater muscle deposition in the carcass. A greater proportion of muscle in the carcass is desirable since the muscle is the edible portion of the carcass. In contrast, the lower performance observed with inclusion levels above 90 g/kg DM can be explained by the reduction in the intake (kg/day) of DM, CP, NFC, and TDN and increasing EE intake (kg/day). Allen et al. [40] reported that high levels of ether extract promote reduced intake and diet acceptability, impacting rumen fermentation and the release of intestinal hormones that act to control satiety. The observed reduction in the intake (kg/day) of nutrients (CP, NFC, and NDT) has a direct impact on animal performance [41,42].

## 5. Conclusions

The replacement of soybean meal and corn ground with sunflower cake at a level of 90 g/kg of DM in the diet of young bulls is recommended because it increases microbial protein synthesis and muscle tissue deposition compared to the control treatment. Consequently, improving the performance, feed efficiency, and carcass traits of the animals.

## Figures and Tables

**Table 1:** Chemical composition of the ingredients used in the experimental diets.

Chemical Composition (g/kg DM)	Tifton-85	Corn Ground	Soybean Meal	Sunflower Cake
Dry matter (g/kg as fed diet)	854	901	879	890
Ash	59.3	12.8	65.8	60.7
Crude protein	78.4	94.9	503	249
NDIN (g/kg CP)	58.9	120	53.4	127
ADIN (g/kg CP)	34.2	3.00	0.40	26.8
Ether extract	13.7	51.4	17.4	162
_ap_Neutral detergent fiber	720	112	103	318
Acid detergent fiber	397	23.2	71.3	232
Nonfibrous carbohydrates	129	729	311	210
Hemicellulose	324	88.5	32.1	86
Cellulose	336	22.6	70.1	165
Acid detergent lignin	60.8	0.71	1.32	67.7
_i_NDF	247	77.2	70.3	176
Total nitrogen fraction (g/kg CP)				
A + B1 + B2	411	880	947	873
B3	555	117	52.6	10
C	34	3	0.4	27
Carbohydrate fractions				
A + B1	195	733	584	589
B2	659	111	102	251
C	146	156	317	160

Abbreviations: NDIN, neutral detergent insoluble nitrogen; ADIN, acid detergent insoluble nitrogen; CP, crude protein; _ap_, corrected for the ash and protein contents; _i_NDF, indigestible neutral detergent fiber.

**Table 2:** Ingredient proportions and chemical composition of the experimental diets.

Variables	Sunflower Cake Level g/kg DM			
	0	90	180	270
Ingredients (g/kg DM)				
Tifton-85 hay	400.0	400.0	400.0	400.0
Corn ground	460.0	408.0	357.0	305.0
Soybean meal	115.0	77.0	38.0	0.0
Sunflower cake	0.0	90.0	180.0	270.0
Urea + ammonia sulfate ^1^	10.0	10.0	10.0	10.0
Mineral mixture ^2^	15.0	15.0	15.0	15.0
Chemical composition (g/kg DM)				
Dry matter (g/kg of the as-fed diet)	882	882	882	882
Ash	52.2	54.5	56.7	59.0
Crude protein	159	158	156	154
NDIN (g/kg CP)	297	300	303	306
ADIN (g/kg CP)	15.1	17.3	19.6	21.8
Ether extract	31.1	42.4	53.6	64.9
_ap_Neutral detergent fiber	351	370	389	408
Acid detergent fiber	178	195	212	229
Nonfibrous carbohydrates	424	394	363	332
Hemicellulose	174	176	178	180
Cellulose	153	164	175	186
Acid detergent lignin	24.8	30.7	36.6	42.5
_i_NDF	142	151	161	170
Total nitrogen fraction (g/kg CP)				
A + B1 + B2	688	685	682	679
B3	282	283	284	285
C	15.1	17.3	19.6	21.8
Carbohydrate fractions (g/kg DM)				
A + B1	613.6	586.3	559.1	532
B2	327	340	353	366
C	59.4	73.7	87.9	102.0

Abbreviations: NDIN, neutral detergent insoluble nitrogen; ADIN, acid detergent insoluble nitrogen; CP, crude protein; _ap_, corrected for the ash and protein contents; _i_NDF, indigestible neutral detergent fiber. ^1^ Mixture of urea and ammonium sulfate in a ratio of 9:1; ^2^ guaranteed levels (for active elements): 210 g of calcium, 163 g of phosphorus, 147 g of sodium, 12 g of sulfur, 3500 mg of copper, 310 mg of cobalt, 20 mg of chromium, 1960 mg of iron, 280 mg of iodine, 3640 mg of manganese, 32 mg of selenium, 9000 mg of zinc, and a maximum of 1630 mg of fluoride.

**Table 3:** Intake, effectively ingested diet composition, and ingestive behavior of young bulls fed diets containing sunflower cake.

Variables	Sunflower Cake (g/kg DM)				SEM	*p*-Value ^1^	
	0.0	90	180	270		Linear	Quadratic
Intake (kg/d)							
Dry matter	11.1	10.8	10.3	10.2	0.08	0.003	0.57
Crude protein	1.91	1.83	1.71	1.66	0.02	0.004	0.79
Ether extract	0.39	0.51	0.63	0.75	0.02	<0.001	0.92
Nonfibrous carbohydrates	4.69	4.32	3.73	3.36	0.10	<0.001	0.99
_ap_Neutral detergent fiber	3.73	3.79	3.83	3.99	0.04	0.99	0.54
Total digestible nutrients	7.33	7.47	7.04	7.18	0.09	0.70	0.94
Intake (g/kg BW)							
Dry matter	25.3	24.3	23.5	23.0	0.05	0.002	0.44
_ap_Neutral detergent fiber	8.50	8.50	8.70	9.00	0.02	0.94	0.68
Effectively ingested diet composition (g/kg DM)							
Dry matter (g/kg as-fed)	765	772	764	770	1.36	0.68	0.66
Crude protein	172	169	166	164	0.80	0.002	0.65
Ether extract	34.8	47.4	60.8	73.4	2.60	<0.001	0.63
Nonfibrous carbohydrates	422	398	362	329	6.34	<0.001	0.11
_ap_Neutral detergent fiber	337	349	372	391	3.87	<0.001	0.12
Ingestive behavior (min/d)							
Eating time	173	190	221	224	7.07	0.014	0.55
Ruminating	370	480	469	453	15.1	<0.001	0.033
Idling	897	770	750	763	19.1	<0.001	0.043
Efficiency rate							
Eating time (kg DM/h)	0.40	0.35	0.29	0.28	0.015	0.042	0.012
Ruminating (kg DM/h)	0.20	0.14	0.14	0.14	0.009	<0.001	0.034
Ruminating (kg NDF/h)	0.68	0.48	0.50	0.54	0.003	<0.001	0.033

Abbreviations: SEM, standard error of the mean; BW, body weight; _ap_, corrected for the ash and protein contents. ^1^ Significance at *p* < 0.05.

**Table 4:** Digestibility, microbial production efficiency, and blood serum metabolites in young bulls fed diets containing sunflower cake.

Variables	Sunflower Cake (g/kg DM)				SEM	*p*-Value ^1^	
	0.0	90	180	270		Linear	Quadratic
Digestibility (g/100 g Ingested)							
Dry matter	62.6	55.9	54.0	52.4	0.83	<0.001	0.11
Crude protein	72.6	72.5	72.4	69.6	0.82	0.66	0.41
Ether extract	80.2	75.4	78.4	69.2	0.92	0.88	0.12
Nonfibrous carbohydrates	79.0	74.5	76.4	77.7	0.11	0.20	0.20
_ap_Neutral detergent fiber	59.2	51.8	51.4	47.0	0.89	<0.001	0.13
Total digestible nutrients	66.2	68.8	68.3	70.7	0.60	0.39	0.88
Nitrogen (N) balance (g/d)							
N-intake	306	293	274	265	4.12	<0.001	0.77
N-fecal excretion	88.2	85.9	83.0	86.0	2.27	0.50	0.57
N-urinary excretion	43.9	38.5	20.2	19.9	1.99	<0.001	0.12
N-total excretion	132	124	103	106	2.65	<0.002	0.12
N-retained	173	169	171	159	3.78	0.92	0.64
N-excretion (g/100 g of N-intake)							
N-fecal excretion	28.8	29.3	30.3	32.5	2.01	0.48	0.56
N-urinary excretion	14.3	13.1	7.37	7.51	1.83	<0.001	0.11
N-total excretion	43.2	42.5	37.7	40.0	2.03	<0.003	<0.001
N-retained	56.5	57.7	62.4	60.0	2.23	<0.001	<0.001
Microbial protein production ^2^	66.0	97.9	98.6	102	0.99	<0.001	0.012
Microbial protein efficiency ^3^	9.0	13.1	14.0	14.2	0.63	<0.001	0.083
Serum metabolite profiles							
BUN (mg/dL)	25.7	23.7	21.1	24.3	0.75	0.051	0.082
Total protein (g/L)	8.91	8.92	8.68	8.56	0.14	0.94	0.83
Albumin (g/L)	3.34	3.66	3.18	3.86	0.13	0.71	0.51
Globulin (g/L)	5.57	5.26	5.49	4.70	0.17	0.81	0.47
A:G ratio ^4^	0.64	0.72	0.61	0.84	0.04	0.58	0.33
Cholesterol (mg/dL)	108	159	181	186	10.3	0.042	0.20
Triglycerides (mg/dL)	104	98.5	99.7	102	2.94	0.51	0.52
AST (U/L)	79.1	69.6	72.9	73.0	2.40	0.26	0.32
ALT (U/L)	39.7	39.9	38.8	36.6	1.41	0.87	0.68
GGT (U/L)	7.44	6.83	6.60	7.98	0.24	0.034	0.023
Total bilirubin (mg/dL)	0.32	0.40	0.41	0.33	0.02	0.21	0.013

Abbreviations: SEM, standard error of the mean; BW, body weight; CP, crude protein; _ap_, corrected for the ash and protein contents; _i_NDF, indigestible neutral detergent fiber. BUN, blood urea nitrogen; AST, aspartate aminotransferase; ALT, alanine aminotransferase; GGT, gamma-glutamyltransferase. ^1^ Significance at *p* < 0.05; ^2^ (g Nmicr production/d); ^3^ (Nmicr production/total digestible nutrients intake); ^4^ A:G ratio, albumin:globulin ratio.

**Table 5:** Performance and carcass traits of young bulls fed sunflower cake.

Variables	Sunflower Cake (g/kg DM)				SEM	*p*-Value ^1^	
	0.0	90	180	270		Linear	Quadratic
Initial BW, kg	374	373	374	377	-	-	-
Final BW, kg	509	530	508	514	8.71	0.68	<0.001
Total weight gain, kg	135	158	134	137	4.43	0.35	<0.001
Average daily gain, kg	1.50	1.75	1.49	1.52	0.05	0.35	<0.001
ADG:DMI ratio, kg/kg	0.13	0.16	0.14	0.15	0.01	0.42	0.018
Hot carcass weight, kg	278	289	273	269	5.47	0.69	<0.001
Cold carcass weight, kg	271	280	265	261	5.22	0.73	<0.001
Cooling loss, g/kg	270	310	281	312	0.15	0.56	0.71
Hot carcass yield, g/kg	546	545	536	524	0.29	0.92	0.29
Cold carcass yield, g/kg	532	528	521	508	0.27	0.93	0.30
Carcass length, cm	140	143	142	143	0.81	0.093	0.16
CCI, kg/cm	1.87	1.99	1.88	1.85	0.37	0.40	0.30
LMA, cm^2^	61.3	61.7	68.9	59.1	1.70	0.153	0.14
SFT, mm	3.54	3.46	3.46	2.88	0.24	0.82	0.61
HH section (g/kg tissue)							
Bone	225	196	208	217	0.54	0.084	0.083
Muscle	530	569	559	524	1.03	0.072	0.053
Fat	245	227	233	259	1.05	0.402	0.31
Muscle/bone ratio	241	301	278	241	0.10	<0.001	0.012
Muscle/fat ratio	227	264	269	221	0.15	0.19	0.16

Abbreviations: SEM, standard error of the mean; BW, body weight; ADG:DMI ratio, average daily gain:dry matter intake ratio; CCI, carcass compactness index; LMA, *Longissimus* muscle area; SFT = subcutaneous fat thickness. ^1^ Significance at *p* < 0.05.

## Data Availability

The data that support the findings of this study are available from the corresponding author upon reasonable request.
